# Analysis of the Frequency-Dependent Vibration Rectification Error in Area-Variation-Based Capacitive MEMS Accelerometers

**DOI:** 10.3390/mi15010065

**Published:** 2023-12-28

**Authors:** Shaolin Zhang, Zhi Li, Qiu Wang, Yuanxia Yang, Yongzhen Wang, Wen He, Jinquan Liu, Liangcheng Tu, Huafeng Liu

**Affiliations:** 1 MOE Key Laboratory of Fundamental Physical Quantities Measurement & Hubei Key Laboratory of Gravitation and Quantum Physics, PGMF and School of Physics, Huazhong University of Science and Technology, Wuhan 430074, China; zhangshaolin@hust.edu.cn (S.Z.); phdlizhi@hust.edu.cn (Z.L.); qiuwang@hust.edu.cn (Q.W.); tuliangch@sysu.edu.cn (L.T.); 2 System Design Institude of Hubei Aerospace Technology Academy, Wuhan 430040, China; yuanxiayang@gmail.com; 3 Scientific and Technological Innovation Center, Beijing 100020, China; yongzhenwang@gmail.com; 4 The State Key Laboratory of Fluid Power and Mechatronic System, Zhejiang University, Hangzhou 310027, China; hewens@zju.edu.cn; 5 MOE Key Laboratory of TianQin Mission, TianQin Research Center for Gravitational Physics & School of Physics and Astronomy, Frontiers Science Center for TianQin, CNSA Research Center for Gravitational Waves, Sun Yat-sen University (Zhuhai Campus), Zhuhai 519082, China

**Keywords:** MEMS, capacitive, accelerometer, second-order nonlinearity, vibration rectification error

## Abstract

The presence of strong ambient vibrations could have a negative impact on applications such as high precision inertial navigation and tilt measurement due to the vibration rectification error (VRE) of the accelerometer. In this paper, we investigate the origins of the VRE using a self-developed MEMS accelerometer equipped with an area-variation-based capacitive displacement transducer. Our findings indicate that the second-order nonlinearity coefficient is dependent on the frequency but the VRE remains constant when the displacement amplitude of the excitation is maintained at a constant level. This frequency dependence of nonlinearity is a result of several factors coupling with each other during signal conversion from acceleration to electrical output signal. These factors include the amplification of the proof mass’s amplitude as the excitation frequency approaches resonance, the nonlinearity in capacitance-displacement conversion at larger displacements caused by the fringing effect, and the offset of the mechanical suspension’s equilibrium point from the null position of the differential capacitance electrodes. Through displacement transducer and damping optimization, the second-order nonlinearity coefficient is greatly reduced from mg/g^2^ to μ g/g^2^.

## 1. Introduction

Accelerometers are devices used to measure acceleration and they have been extensively utilized in various fields such as consumer electronics, automobile manufacturing, and industry applications that involve inertial measurements. In certain scenarios, strong vibrations at various frequencies in the environment can cause distortion of the accelerometer output while acceleration measurement of high precision is sought [1,2,3]. The vibration rectification error (VRE) refers to the phenomenon where alternating current (AC) vibrations are rectified into direct current (DC) output of the accelerometer [4,5]. It is manifested as a significant shift in the DC output, even when there is originally no DC input present [6,7]. This kind of error can have a significant impact, particularly when the DC output of the accelerometer is used to determine displacement by double integration over time. For example, DC errors could lead to an intolerant cumulative error on displacement over extended periods for inertial navigation [8]. Taking the inclination measurement [9] based on measuring gravitational acceleration as another example, any DC shifting of the accelerometer output can be incorrectly interpreted as changes in inclination, which would cause over-compensation in platform stabilization or drill mast alignment [2].

Accelerometer nonlinearity has been identified as the primary cause of VRE [10,11,12,13]. In order to suppress the VRE, extensive studies have been conducted on the origins of the VRE. In pendulous accelerometers, the deflection angle of the pendulum is linked to the rectification error, as smaller angles correspond to a smaller rectification error [14]. Additionally, the rectification error can be attributed to asymmetrical behavior of the residual rotational stiffness and the damping torque [15]. Both open-loop MEMS capacitive accelerometers and the closed-loop ones exhibit a rectification phenomenon [16,17]. In a closed-loop capacitive accelerometer with the comb-finger sensing scheme, the gas-spring damping generating a non-zero time-averaged force on the proof mass and the voltage-related nonlinear rebalance force are considered the main causes of the VRE [6]. While most studies have focused on the error effects of individual steps in the signal transduction process, there are few that address the VRE arising from coupling between modules such as the spring-mass mechanical suspension and the displacement detection circuit.

This paper investigates the vibration rectification phenomenon using a self-developed open-loop MEMS capacitive accelerometer. Firstly, we introduce the operating principle and design of the proposed accelerometer, including the spring-mass mechanical suspension, area-variation-based capacitive displacement transducer, and the fabrication process. Secondly, we analyze the main mechanism underlying the VRE theoretically, specifically focusing on the imperfections of each step involved in the conversion from input acceleration into electrical output and possible coupling between steps. Finally, we conduct tests on nonlinearity of MEMS accelerometers based on their response to varying excitation frequency with a given amplitude. Through analysis of the experimental results, we aim to provide insights into the generation of the VRE and reduce the VRE of the optimized accelerometer.

## 2. Description of the MEMS Accelerometer

**Figure 1:**
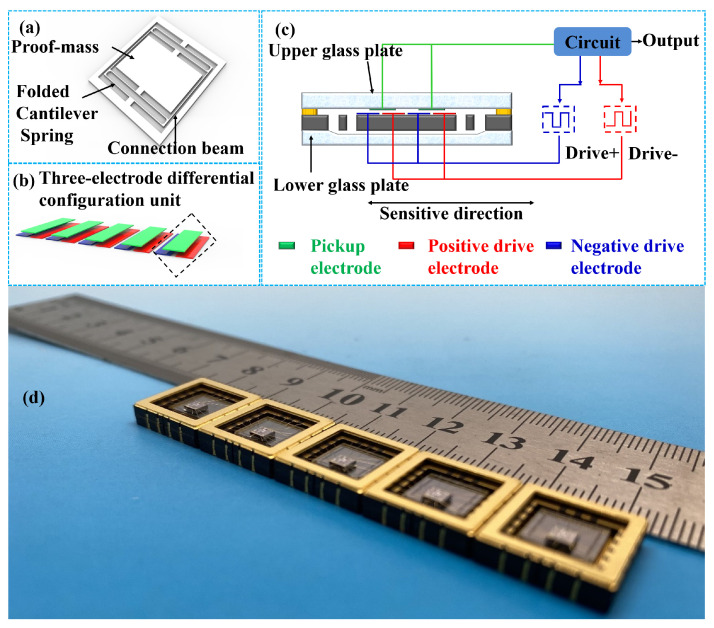
(**a**) The structure of the MEMS suspension; (**b**) The configuration of the area-variation-based capacitive displacement transducers (CDT); (**c**) The schematic diagram of the proposed accelerometer; (**d**) MEMS die packaged in a ceramic chip carrier.

In this paper, a capacitive MEMS accelerometer self-developed in house is employed to investigate the VRE effect. As shown in Figure 1, its overall configuration includes three parts: mechanical suspension, capacitance displacement detection, and electronic signal conversion.

The MEMS suspension moves laterally, and it is sensitive to in-plane motions, as shown in Figure 1a. The mechanical suspension is overall bilaterally symmetrical. In its simplest form, the suspension consists of two folded cantilever springs on either side of a proof mass, which is connected to an external fixed frame. The use of folded cantilever springs could help to improve linearity. The springs on both sides of the proof mass are monolithically connected by intermediate beams to reduce the cross-sensitivity. The capacitance of the area-variation-based capacitive displacement transducers (CDT) changes with the variation of displacement when there is an acceleration input along the sensitive direction, as shown in Figure 1b. The adjacent drive electrodes on the proof mass are driven by carrier signals with a phase difference of 180^∘^, while the upper glass substrate is composed of pickup electrodes picking up the differential signal. Each group of two drive electrodes and one pickup electrode forms a three-electrode differential capacitance unit, or a differential electrode pair. To increase the displacement-to-capacitance gain, an array including *N* pairs of the differential electrode units is utilized. The detection circuit responsible for capacitance-to-voltage conversion generates the carrier waves and feeds them to the drive electrodes. The resulting voltage signal is proportional to the capacitance and, thus, to the input acceleration, as shown in Figure 1c. Figure 1d shows the MEMS dies packaged in a ceramic chip carrier.

**Figure 2:**
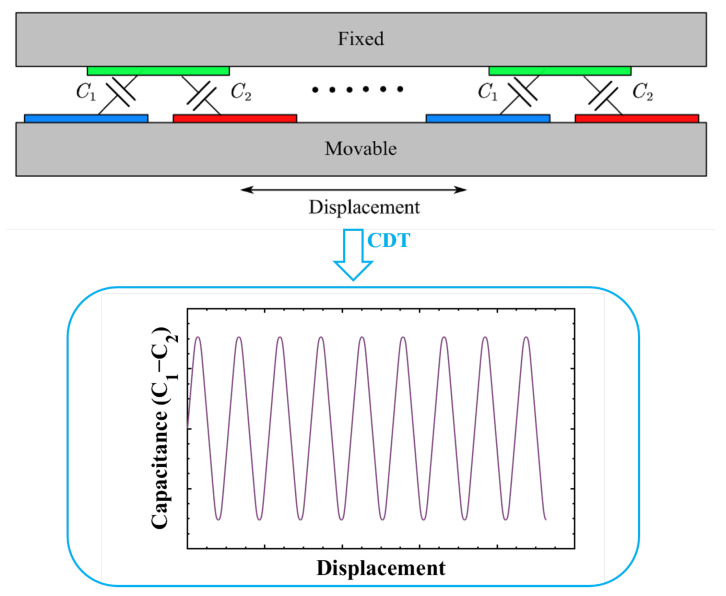
The periodic relationship between the capacitance and the displacement of the proof mass.

Practically, the pair number of the capacitance array cannot be increased excessively. The differential capacitance between the differential electrode pair is plotted as a function of the proof mass displacement, as shown in Figure 2. The differential capacitance ( $C_{1}-C_{2}$ ) of the CDT changes periodically with the displacement. It is desirable to keep the proof mass traveling within a monotonic interval of the capacitance-displacement curve over the entire operational range where the capacitance has the most sensitive and linear relationship with the displacement. Nonlinearity would dramatically rise up beyond this interval and the sensitivity would become low on approach to the periodic inflection point. Particularly for an open loop accelerometer, the width of electrodes should be significantly large compared with the maximum displacement. The proof mass displacement of the mechanical suspension might contribute to the nonlinearity by coupling with imperfections of the capacitance electrodes, which is one important topic of this paper.

**Figure 3:**
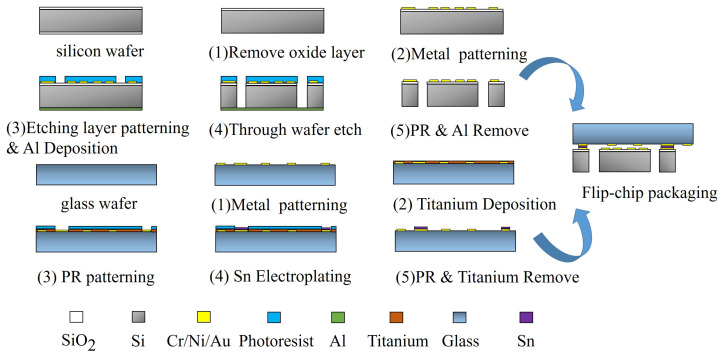
The fabrication process flow of the proposed accelerometer chip.

The fabrication of the proposed MEMS accelerometer is inherited from previous work [18,19].The fabrication process flow of the silicon substrate made of single-crystal silicon and the upper glass plate made of Borofloat 33 is shown in Figure 3. They are fabricated separately and then aligned and packaged.

The fabrication process of the proposed accelerometer is composed of the following steps. Firstly, a silicon wafer with double side oxidation was prepared. The backside oxidation layer was removed by reactive ion etching (RIE), and the front side window was patterned through photolithography. The Metal layer of Cr (20 nm)/Ni (80 nm)/Au (400 nm) was deposited by E-beam evaporation and patterned by lift-off technology to form the electrodes. The spring-mass structure mask was formed with a 15 μ m thick AZ9260 photoresist (Merck, Whitehouse Station, NJ, USA) that was spin-coated and patterned next. Then, a 300-nm-thickness aluminum layer was deposited on the backside of the wafer. Another wafer was attached to the bottom of the device wafer with a 2- μ m-thickness photoresist layer. Then, the Oxford Instrument (Oxford Instrument, Abingdon, UK) inductively coupled plasma (ICP) system was used to conduct the DRIE through-wafer-etching process. The silicon-based spring-mass structure with a thickness of 500 μ m was released and free to move after photoresist and aluminum stripping. Lastly, each individual die was singulated from the wafer using dicing-free technology.

The upper glass plate of the proposed MEMS sensor was fabricated based on a 500- μ m-thickness glass wafer. Firstly, the metal features of Cr (20 nm)/Ni (80 nm)/Au (400 nm) were fabricated by evaporation and lift-off technologies. Secondly, a titanium layer of 100 nm in thickness was deposited and patterned to form a mask. A tin electroplating process was conducted to form the air gaps and packaging features with a thickness of 6 μ m. Lastly, the photoresist and titanium layers were stripped. The glass wafer was then diced into individual dies. The silicon-based spring-mass layer and the glass-based upper plate were flip-chip packaged by Au-Sn thermo-compression bonding at a temperature of 280 ^∘^C for 30 min.

Then, a lower glass cover plate with a cavity was bonded to the backside of the silicon layer to play an important role in protecting the movable structure, forming a MEMS die. The fabricated MEMS die was packaged in a ceramic chip carrier. Electrical connections were conducted by gold-wire bonding. The carrier lid was sealed under a nitrogen environment, as shown in Figure 1d.

## 3. Model of the Accelerometer and Its VRE

In this section, the output model equation of the accelerometer with respect to the input acceleration is provided. Then, the VRE based on this model equation is derived. In order to find potential nonlinearity error effects, we analyze each step of the signal conversion process from acceleration to final electric output and coupling between steps.

### 3.1. Output Model of the Accelerometer and Its VRE

The output model equation of the accelerometer can be represented as a simplified series that mathematically relates the accelerometer output to the components of applied acceleration [20]. In this study, our focus is on the accelerometer response to acceleration along the input axis. Hence, we neglect error effects related to acceleration along the cross axes, resulting in a final equation that only includes terms of our interest. This equation can be reduced to:

(1) $$E=K_{1}\left\{K_{0}+a_{i}+K_{2}a_{i}^{2}+K_{3}a_{i}^{3}\right\},$$

where *E* denotes the accelerometer output (volts, simplified as V), and $K_{0},K_{1},K_{2},K_{3}$ denote the bias (g, local value of gravity), accelerometer scale factor (V/g), the second-order nonlinearity (g/g^2^) and the third-order nonlinearity (g/g^3^), respectively.

Suppose that the accelerometer is subjected to a vibration signal $a_{i}=Asin\left(\omega t\right)$ , where *A* and ω represent the amplitude and angular frequency of the vibration, respectively. According to Equation (1), we can deduce the DC component of the accelerometer output as

(2) $$\bar{E}=K_{1}K_{0}+\frac{1}{2}K_{1}K_{2}A^{2}.$$

According to the definition, the VRE induced by the second-order nonlinearity can be written as

(3) $$VRE=\frac{\bar{E}-K_{1}K_{0}}{K_{1}}=\frac{1}{2}K_{2}A^{2}.$$

### 3.2. Analysis of Accelerometer’s Signal Conversion Process by Steps and Origin of Second-Order Nonlinearity

In this section, we build up the relationship between the coefficients of the accelerometer’s model equation and the signal conversion process from acceleration input to electrical output. It will help to find the origins of error terms and optimize the design to meet the specific requirements.

As shown in Figure 1, the signal conversion of our open loop accelerometer is composed of three consecutive steps: acceleration-to-displacement conversion, displacement-to-capacitance conversion, and capacitance-to-voltage conversion. A lock-in amplifier (MFLI 5 MHz, Zurich Instruments, Zürich, Switzerland) is used in the experiment setup to ensure that capacitance-voltage conversion $H_{C-E}$ can be considered as highly linear. Hence, $H_{C-E}$ can be simply taken as a constant. We will focus on analysis of the acceleration-to-displacement conversion and the displacement-to-capacitance conversion step, in sequence.

It is known that the response of the mechanical suspension can be described over a broad frequency range as a linear second-order system. The amplitude transfer function $H_{A-X}$ of acceleration-to-displacement conversion is written as

(4) $$H_{A-X}\left(\omega \right)=\frac{X}{A}=\frac{1}{\sqrt{{\left({\omega_{0}}^{2}-\omega^{2}\right)}^{2}+{\left(2\zeta \omega_{0}\omega \right)}^{2}}}.$$

where ζ denotes the damping ratio, $\omega_{0}$ and ω denote the natural resonance angular frequency of the mechanical suspension and the angular frequency of the excitation signal, respectively. Due to the bilaterally symmetrical configuration of the suspension as shown in Figure 1a, the spring elasticity and the gas damping on either side of the proof mass is also bilaterally symmetrical. Hence, the second-order nonlinearity of $H_{A-X}$ should be completely cancelled out with an ideally-fabricated mechanical suspension. The finite element simulation results show that the nonlinearity of the mechanical suspension is minor using a properly designed structure such as the folded beam, as shown in Figure 1a. Even if imperfection of structures occurs during fabrication, for example, that the width of the beam on one side is different from that of the opposite side by an error of 2 μ m, the second-order nonlinearity coefficient does not exceed 0.1 μ g/g^2^. An input acceleration of 60 g is given for simulation here. Therefore, the mechanical suspension response of our accelerometer can be described very well with Equation (4).

When $\omega \ll \omega_{0}$ , the displacement amplitude *X* of the proof mass can be approximately taken as

(5) $$H_{A-X}\left(\omega \right)=\frac{1}{\omega_{0}^{2}}=H_{A-X}\left(0\right).$$

The conversion factor $1/\omega_{0}^{2}$ is a constant, the same as that for a DC acceleration input, in this frequency range.

When the angular frequency ω of the input acceleration is not much smaller than the resonance $\omega_{0}$ , the displacement response shows dependence on the frequency. Especially, when the input acceleration frequency is coincident with the resonance, the proof mass displacement amplitude will be magnified *Q* times. *Q* is the quality factor of mechanical suspension and Q=1/2ζ .

**Figure 4:**
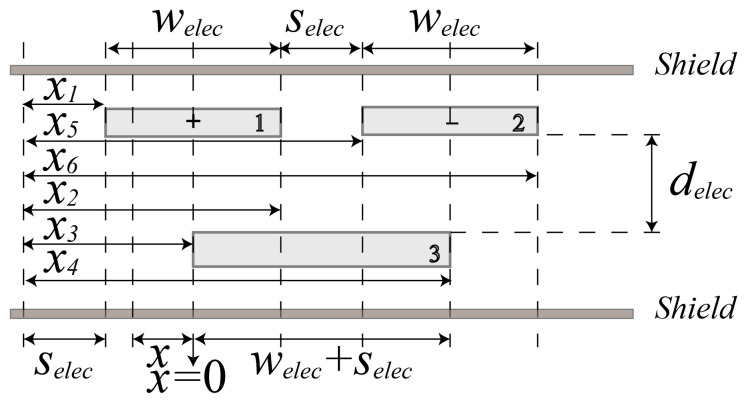
Configuration of a differential capacitance unit with three electrodes.

Figure 4 shows the details of one three-electrode differential capacitance unit in Figure 1b. The two drive electrodes of the capacitance unit have a width of $w_{\mathrm{elec}}$ , a length of $L_{\mathrm{elec}}$ and a distance of $s_{\mathrm{elec}}$ between their neighboring edges, the pickup electrode has a width of $w_{\mathrm{elec}}+s_{\mathrm{elec}}$ and a length of $L_{\mathrm{elec}}$ , and the gap between the drive and pickup electrodes is $d_{\mathrm{elec}}$ . Regarding the capacitance of a typical overlapped parallel-plate capacitor, since the dimensions of its electrodes are not infinitely large relative to the gap in between the opposite parallel electrodes, the fringing field could play a significant role when the pickup electrodes deviate from the central position between two opposite adjacent drive electrodes under large acceleration. The capacitance cannot be simply treated as proportional to the facing area any more. We use the Heerens’ model [21] in order to take the fringing effect into account. The capacitance $C_{13}$ between electrode 1 and 2, and $C_{23}$ between electrode 2 and 3 are expressed in relationship to the relative displacement *x* between electrodes as

(6) $$\begin{array}{cc} C_{13} & =\frac{-\varepsilon_{0}\varepsilon_{r}L_{\mathrm{elec}}}{\pi }\ln\left[\frac{\cosh\left[\frac{\pi \left(x_{1}-x_{3}\right)}{2d_{\mathrm{elec}}}\right]\cosh\left[\frac{\pi \left(x_{2}-x_{4}\right)}{2d_{\mathrm{elec}}}\right]}{\cosh\left[\frac{\pi \left(x_{2}-x_{3}\right)}{2d_{\mathrm{elec}}}\right]\cosh\left[\frac{\pi \left(x_{1}-x_{4}\right)}{2d_{\mathrm{elec}}}\right]}\right]\\ \; & =\frac{-\varepsilon_{0}\varepsilon_{r}L_{\mathrm{elec}}}{\pi }\ln\left[\frac{\cosh\left[\frac{-\pi \left(x+\frac{w_{\mathrm{elec}}}{2}\right)}{2d_{\mathrm{elec}}}\right]\cosh\left[\frac{-\pi \left(x+\frac{w_{\mathrm{elec}}}{2}+s_{\mathrm{elec}}\right)}{2d_{\mathrm{elec}}}\right]}{\cosh\left[\frac{-\pi \left(x-\frac{w_{\mathrm{elec}}}{2}\right)}{2d_{\mathrm{elec}}}\right]\cosh\left[\frac{-\pi \left(\frac{3w_{\mathrm{elec}}}{2}+s_{\mathrm{elec}}+x\right)}{2d_{\mathrm{elec}}}\right]}\right]. \end{array}$$

(7) $$\begin{array}{cc} C_{23} & =\frac{-\varepsilon_{0}\varepsilon_{r}L_{elec}}{\pi }\ln\left[\frac{\cosh\left[\frac{\pi \left(x_{5}-x_{3}\right)}{2d_{\mathrm{elec}}}\right]\cosh\left[\frac{\pi \left(x_{6}-x_{4}\right)}{2d_{\mathrm{elec}}}\right]}{\cosh\left[\frac{\pi \left(x_{6}-x_{3}\right)}{2d_{\mathrm{elec}}}\right]\cosh\left[\frac{\pi \left(x_{5}-x_{4}\right)}{2d_{\mathrm{elec}}}\right]}\right]\\ \; & =\frac{-\varepsilon_{0}\varepsilon_{r}L_{\mathrm{elec}}}{\pi }\ln\left[\frac{\cosh\left[\frac{-\pi \left(x-\frac{w_{\mathrm{elec}}}{2}-s_{\mathrm{elec}}\right)}{2d_{\mathrm{elec}}}\right]\cosh\left[\frac{-\pi \left(x-\frac{w_{\mathrm{elec}}}{2}\right)}{2d_{\mathrm{elec}}}\right]}{\cosh\left[\frac{-\pi \left(x-\frac{3w_{\mathrm{elec}}}{2}-s_{\mathrm{elec}}\right)}{2d_{\mathrm{elec}}}\right]\cosh\left[\frac{-\pi \left(x+\frac{w_{\mathrm{elec}}}{2}\right)}{2d_{\mathrm{elec}}}\right]}\right]. \end{array}$$

where $x_{1}$ , $x_{2}$ , $x_{3}$ , $x_{4}$ , $x_{5}$ , $x_{6}$ respectively denote the distance from the left or right edge of the three electrodes in this unit to the right edge of the drive electrode in the left neighboring unit. Note that the origin of *x* is a position where the pickup electrode is laterally centered in two adjacent drive electrodes of the opposite substrate, where the differential capacitance is zero. Consequently, the differential capacitance ( $C_{13}-C_{23}$ ) varying with proof mass displacement can be obtained.

In order to find the corresponding relationship between the model Equation (1) and the electrode relative displacement, we have to treat the conversion relationship of every conversion step in a linear form as $H_{X-C}=C/X=h_{1}$ or in a polynomial form between *C* and *X*. Here, *C* is the overall differential capacitance of the differential capacitance array including *N* units. Considering the nonlinearity of this step, the displacement-to-capacitance conversion is better described in a cubic polynomial form as

(8) $$C=h_{1}X+h_{2}X^{2}+h_{3}X^{3}.$$

The coefficients of $h_{1}$ , $h_{2}$ and $h_{3}$ can be acquired by fitting Equation (8) with the calculated C−X relationship according to Equations (6) and (7) using the method of least squares.

**Figure 5:**
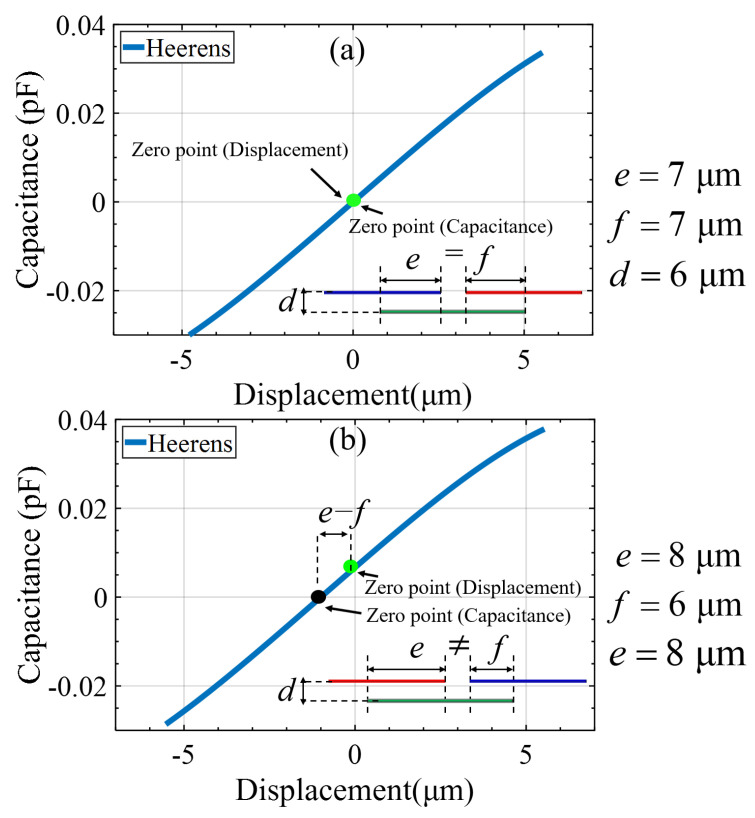
Displacement to capacitance conversion with the consideration of the fringing field. (**a**) The pickup electrodes are originally centered in two adjacent drive electrodes, or vice verse; (**b**) The original position of the electrodes is not centered relative to each other.

Figure 5a illustrates the calculated capacitance-displacement curve for a specific configuration of the capacitance array in an interval around the “zero” point. It can be seen that the capacitance deviates from linear dependence on the displacement when the proof mass is far away from the “zero” point. In this case, there would be only odd-order nonlinearity when the drive electrodes on the proof mass is symmetrically vibrating around the zero capacitance point due to the central symmetry of the relationship. However, the even-order nonlinearity would be introduced when the proof mass is vibrating around a point other than the zero-capacitance point, as shown in Figure 5b. This situation could be caused when there is an imperfect misalignment of opposite electrodes during flip-chip packaging, as shown in the last fabrication step of Figure 3. These interpretations are made by some authors [22,23,24,25].

Therefore, the second-order nonlinearity is deduced to directly come from the fringing effect of the displacement-capacitance conversion step coupling with the packaging misalignment. The frequency dependence of the acceleration-to-displacement conversion step would lead to frequency-dependent nonlinearity via the displacement-capacitance conversion step.

### 3.3. Nonlinearity Analysis in Terms of the Model Equation

We can relate the scale factor in Equation (1) to every conversion step as

(9) $$K_{1}\left(\omega \right)=H_{C-E}h_{1}H_{A-X}\left(\omega \right).$$

Practically, when the frequency of an input acceleration is far below the bandwidth, the scale factor is approximately constantly equal to the its value at DC as

(10) $$K_{1_{\mathrm{DC}}}\approx H_{C-E}h_{1}/\omega_{0}^{2}.$$

Concerning the second-order nonlinearity coefficient, when an input acceleration comprises components whose frequencies are not far below the bandwidth, the response of the accelerometer would become complicated to describe in a simple form. In this study, we simply consider the situation that the accelerometer is excited with single frequency vibration. And then a second-order nonlinearity term can be written as

(11) $$K_{2}\left(\omega \right)=h_{2}H_{A-X}\left(\omega \right)/h_{1}.$$

It can be seen that both the scale factor and the nonlinearity coefficient is frequency-dependent, which will make it challenging to describe the response of the accelerometer when it is used to measure time-varying acceleration involving multi-frequency components. Nevertheless, when the frequency of an input acceleration is far below the bandwidth, we can simply take $K_{2}$ as a constant, which is

(12) $$K_{2_{\mathrm{DC}}}=h_{2}/h_{1}/\omega_{0}^{2}.$$

Based on dimensions of the capacitance unit configuration given in Figure 5 and the corresponding analysis, we can deduce the second-order nonlinearity coefficient as an exemplary case. Suppose that the accelerometer is subjected to vibrational excitation with any frequency far below the resonance frequency, say 1160 Hz, and an amplitude of 10 g, the second-order nonlinearity coefficient is expected to be approximately 1.46 mg/g^2^.

The VRE is also frequency-dependent according to Equation (3) as follows

(13) $$VRE\left(\omega \right)=\frac{1}{2}K_{2}A^{2}=\frac{1}{2}K_{2_{\mathrm{DC}}}{\left(\frac{H_{A-X}\left(\omega \right)}{H_{A-X}\left(0\right)}A\right)}^{2}=\frac{1}{2}\left(K_{2_{\mathrm{DC}}}\frac{K_{1}{\left(\omega \right)}^{2}}{{K_{l_{\mathrm{DC}}}}^{2}}\right)A^{2}.$$

Hence, we have

(14) $$K_{2}=K_{2_{\mathrm{DC}}}\frac{K_{1}{\left(\omega \right)}^{2}}{{K_{1_{\mathrm{DC}}}}^{2}}.$$

Based on the analysis above, coupling between these first two signal conversion steps indeed brings about a frequency-dependence nonlinearity effect. Even if the input acceleration has the same amplitude, the VRE could change since the displacement amplitude of the proof mass would change when the frequency is not far below the resonance in the acceleration-to-displacement step. Particularly, the magnified displacement amplitude around the resonance of a high Q mechanical suspension could indirectly lead to a magnified VRE of the accelerometer. Therefore, both the configuration of the mechanical suspension and the capacitor must be taken into account in order to balance between the accelerometer’s linearity and other properties such as sensitivity, bandwidth, and measurement range. In order to reduce the VRE, we know from these analyses that various means are optional for optimizing the accelerometer performance. For example, we can either reduce Q to suppress the potential nonlinearity magnification at the resonance, or simply increase the resonance frequency of the mechanical suspension to reduce the overall displacement response, or increase the width of the electrode to reduce the fringing effect of capacitance.

## 4. Experiments and Results

### 4.1. Test Method and Setup

**Figure 6:**
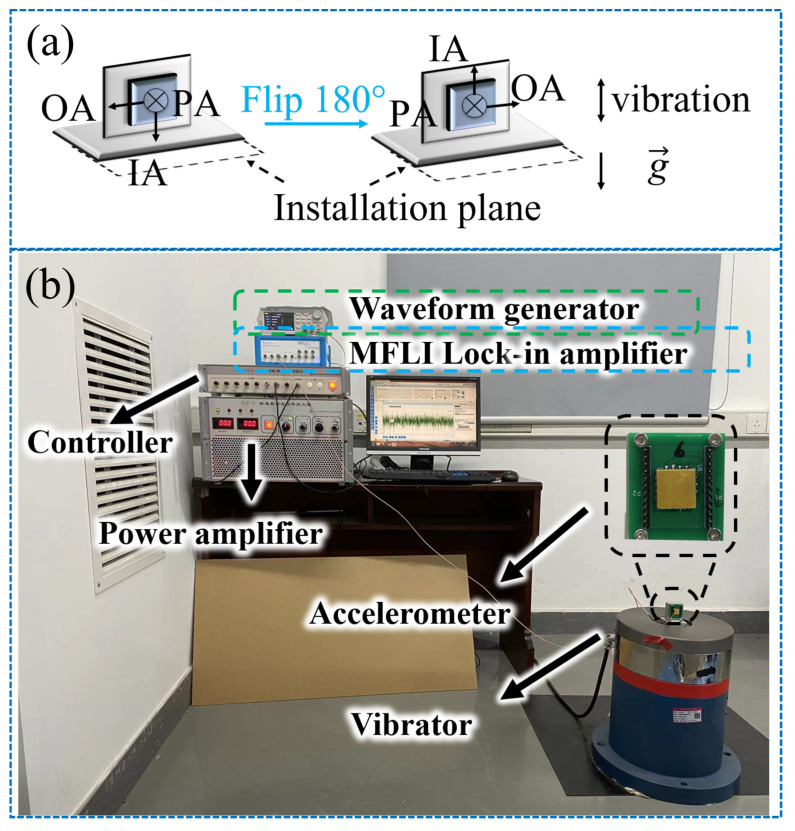
(**a**) Mounting configurations of the accelerometer on the vertically oriented vibrator. (**b**) Accelerometers mounted on the fixture attached to the face plate of a vibrator.

The test was carried out on a shaking table with two mounting configurations (respectively denoted as $M_{1}$ and $M_{2}$ ) of the accelerometer under test, where the input axis (IA) of the accelerometer was parallel or antiparallel to the direction of gravity, as shown in Figure 6a. The configurations can separate a second-order nonlinearity coefficient and a third-order one [20].

Given vibration $a_{i}=Asin\left(\omega t\right)$ and the gravity component along the input axis is written as $g_{j}$ where *j* represents the mounting configuration $M_{1}$ or $M_{2}$ , the input acceleration is $a+g_{j}$ . The average value of the first-, second- and third-power of the input acceleration are respectively deduced to be

(15) $$\begin{array}{cc} \bar{\left(a+g_{j}\right)} & =g_{j},\\ \bar{{\left(a+g_{j}\right)}^{2}} & =\frac{A^{2}}{2}+g_{j}^{2},\\ \bar{{\left(a+g_{j}\right)}^{3}} & =\frac{3A^{2}g_{j}}{2}+g_{j}^{3}. \end{array}$$

where $g_{j}=g_{M_{1}}=-1$ g when the input axis is orientated along the same direction as gravity and $g_{j}=g_{M_{2}}=1$ g along the opposite direction. The accelerometer average outputs $\bar{E_{M_{1}}}$ and $\bar{E_{M_{2}}}$ for the given configurations $M_{1}$ and $M_{2}$ are derived to be

(16) $$\begin{array}{cc} \; & \bar{E_{M_{1}}}=K_{1}\left(K_{0}-g+g^{2}-g^{3}+K_{2}\frac{A^{2}}{2}+K_{3}\frac{3A^{2}g}{2}\right),\\ \; & \bar{E_{M_{2}}}=K_{1}\left(K_{0}+g+g^{2}+g^{3}+K_{2}\frac{A^{2}}{2}-K_{3}\frac{3A^{2}g}{2}\right). \end{array}$$

And then, the DC variation in the accelerometer output is

(17) $$\begin{array}{cc} \; & \Delta \bar{E_{M_{1}}}=K_{2}\frac{A^{2}}{2}+K_{3}\frac{3A^{2}g}{2},\\ \; & \Delta \bar{E_{M_{2}}}=K_{2}\frac{A^{2}}{2}-K_{3}\frac{3A^{2}g}{2}. \end{array}$$

The vibration rectification error corresponding to the second-order nonlinearity is

(18) $$\begin{array}{cc} VRE & =K_{2}\frac{A^{2}}{2}=\frac{\Delta \bar{E_{M_{1}}}+\Delta \bar{E_{M_{2}}}}{2},\\ K_{2} & =\frac{\Delta \bar{E_{M_{1}}}+\Delta \bar{E_{M_{2}}}}{A^{2}}. \end{array}$$

During testing, a special procedure was followed to obtain the DC shift due to vibrational excitation. Considering that the bias of the accelerometer could also drift under influences other than vibration, an interval in the “vibration off” state was set on purpose between the “vibration on” states. In the “vibration on” state, the accelerometer output was recorded under the vibration excitation with the preset magnitude and frequency. In the “vibration off” state, the accelerometer output was also recorded but without vibration excitation, simply for the sake of tracking the bias drift. The DC shift caused during the vibration state was always measured in reference to the bias during the “vibration off” interval next to it.

The experiment was set up with a medium frequency vibration calibration system developed by the institute of manufacturing engineering and automation, Zhejiang University. As shown in Figure 6b, accelerometers were mounted on a fixture attached to the face plate of the vibrator. Data was acquired continuously at a sampling rate of 10 kHz to prevent aliasing.

### 4.2. Preliminary Test of Accelerometer Nonlinearity

The fixture was designed carefully to ensure its resonance far away from the excitation frequency of interest. The resonance frequency of the first mode of the fixture with finite element analysis (FEA) simulation was 5 kHz.

As known, the closer the frequency of input acceleration to the resonance frequency, the greater the displacement of the mass. There is a risk of the mechanical structure of the accelerometer being damaged when it is strongly excited near the resonance. Hence, the frequency response of the accelerometer was preliminarily tested with weak excitation, for the sake of selecting the proper frequency and amplitude of excitation during the nonlinearity coefficient test in the subsequent steps. Three accelerometers were tested for their frequency response. The resonance frequency and the capacitor parameters of the accelerometers are summarized in Table 1. The original position offset of the pickup electrodes was measured using an optical microscope before the accelerometer was ultimately sealed, and the distance between pickup electrodes and the drive electrodes was measured by SEM.

With the frequency response known, the VRE was measured at a series of amplitudes and frequencies covering the resonance. According to Equations (13) and (14), the second-order nonlinearity coefficient is frequency dependent but the VRE could be constant at different frequencies if the displacement amplitude of the proof mass is kept constant. Hence, the acceleration amplitudes were varied for different excitation frequencies in the test while the displacement amplitude of the proof mass was intentionally kept constant. Tests of our accelerometers were carried out with a series of frequencies and acceleration amplitudes summarized in Table 2.

**Figure 7:**
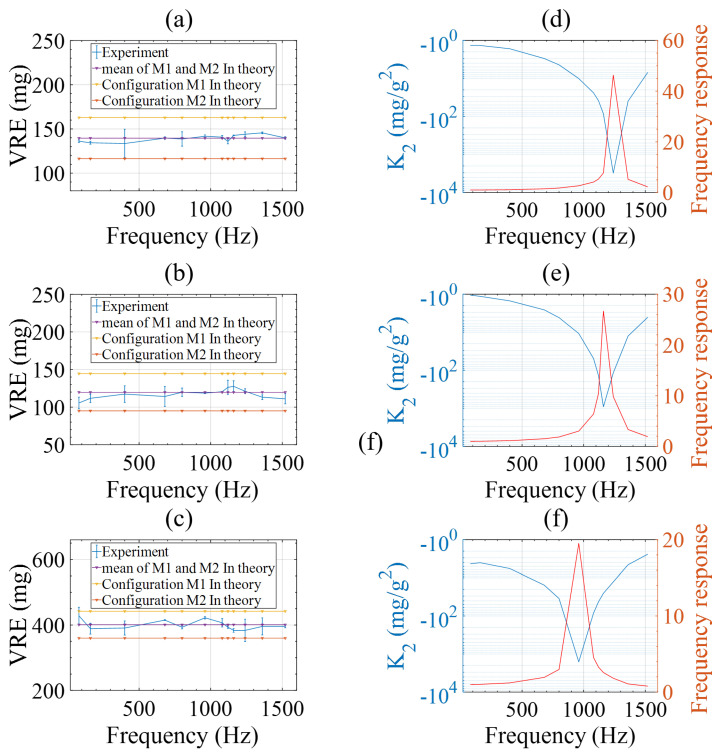
Experiment results of the three tested accelerometers. (**a**–**c**) Vibration-induced rectification error excited at a constant displacement amplitude and varying frequencies; (**d**–**f**) Calculated second-order nonlinearity coefficient and the frequency response.

The corresponding results for the three accelerometers are depicted in Figure 7a–c. Notably, the VRE remained consistent across all frequencies as expected, since the displacement amplitude does not change with varying frequency. Since the accelerometers were installed with their input axis along or opposite to the gravity direction, gravitational acceleration of ±1 g caused different displacement between the pickup and drive electrodes in the two mounting configurations $M_{1}$ and $M_{2}$ . Their DC shifts and the average value were calculated according to the model described in Section 3 using the accelerometer parameters in Table 1 and experimental conditions in Table 2. The results are plotted as three horizontal lines respectively in Figure 7a–c. It can be seen that the theoretical calculation results agree well with the experimental ones.

According to Equation (11), the second-order nonlinearity coefficient $K_{2}$ is deduced and respectively depicted in Figure 7d–f. It is observed for all the three accelerometers that larger $K_{2}$ occurred upon approach to the resonant frequency.

### 4.3. Improvement of Accelerometer Nonlinearity

According to the mechanism verified so far, various measures can be adopted to suppress the nonlinearity of the accelerometer. For example, it is optional to reduce the Q-factor of accelerometers to a proper value such as 0.707 [26,27,28,29,30] to prevent nonlinearity deterioration stimulated at resonance. We chose to enlarge the drive electrode width by a few times, and simultaneously lower the Q-factor by one order of magnitude.

The modeling and experimental results show the fringing effect of a narrow electrode can deteriorate the nonlinearity of accelerometers, while narrow electrodes can favor the sensitivity enhancement of the displacement-to-capacitance conversion. Calculation shows that increasing the drive electrode width from 14 μ m to 42 μ m can suppress $K_{2}$ from mg/g^2^ to μ g/g^2^. The price of electrode width increase is that the displacement-to-capacitance sensitivity is influenced, unless the number of capacitance units remains unchanged by increasing the overall size of the capacitance array. Nevertheless, the influence could be acceptable since the nonlinearity was approximately suppressed by three orders of magnitude, while the electrode width was reduced only by three times. For the maximum displacement corresponding to the operational range of the accelerometer, there is a critical value of the drive electrode width beyond which the second-order nonlinearity coefficient will deteriorate rapidly. The optimized parameters of the accelerometers are shown in Table 3. Furthermore, we sealed the accelerometer die with a narrower gap between the backside of the silicon layer and a lower glass cover plate. The gap narrowed down from an original value of tens of microns to a more stringently controlled value of approximately six microns implemented by Au-Au eutectic bonding. The Q-factor decreased from dozens to less than three, largely mitigating the nonlinearity amplification at resonance.

**Figure 8:**
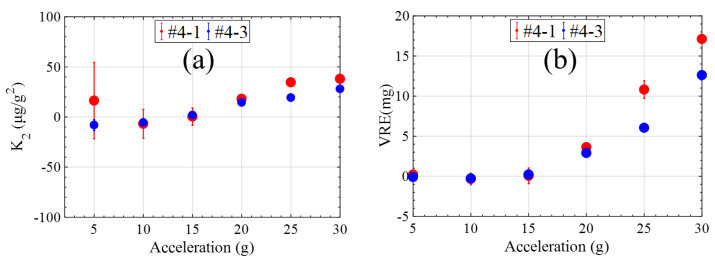
The improvement of the second-order nonlinearity coefficient and VRE. (**a**) The improvement of the second-order nonlinearity. (**b**) The improvement of VRE.

The sinusoidal vibration was chosen to run at constant amplitudes of 5 g, 10 g, 15 g, 20 g, 25 g and 30 g separately with the frequency 160 Hz. The results of the experiment are shown in Figure 8. The second-order nonlinearity coefficient is small. The VRE performance comparisons of the optimized accelerometer with other MEMS accelerometers are listed in Table 4. In terms of VRE performance, the optimized accelerometer in this work had a lower VRE at sinusoidal vibration amplitudes of 10 g, 20 g and 30 g.

## 5. Discussion

The analyses and tests show that the second-order nonlinearity coefficient of our open loop accelerometer is frequency dependent. The dependence can be explained by coupling between the frequency response of the spring-mass mechanical suspension and nonlinear displacement-capacitance conversion. When the excitation frequency approaches the resonance, the input acceleration magnitude would lead to a magnified displacement with an underdamped mechanical suspension. Increased displacement would cause a larger VRE. In order to design a proper accelerometer according to the application requirements, many accelerometer parameters may be taken into account. The area-variation-based capacitance transducer is commonly believed to be more linearly dependent on the displacement than the gap-variation-based one, but one should be cautious to make a tradeoff between increasing sensitivity by using a large number of electrodes and deteriorating nonlinearity by using a narrow electrode. While critically damping the mechanical suspension is a conventional solution requiring extra efforts in manufacturing, underdamped mechanical suspension may still be acceptable in terms of nonlinearity for an open-loop accelerometer if the displacement-capacitance conversion is sufficiently linear by choosing proper configurations of capacitance electrodes.

## 6. Conclusions

In this paper, the mechanism of a VRE of an open-loop capacitive MEMS accelerometer was analyzed first, and then tests were carried out on a medium frequency vibrator to verify the mechanism. The experimental results of three accelerometers showed that the VRE remained constant when the displacement of the proof mass was intentionally kept unchanged at various frequencies. The fringing effect of the differential capacitance unit was primarily responsible for the second-order nonlinearity by coupling with electrode misalignment, and the magnification of the proof mass’s displacement on approach to the resonance will deteriorate the nonlinearity. An optimization strategy involving enlarging the electrode width and increasing the damping was implemented and discussed. As an example, the nonlinearity was greatly decreased from mg/g^2^ to μ g/g^2^ after enlarging the width of electrode by three times, as expected. The results described in this article could provide clues to VRE optimization of open-loop MEMS capacitive accelerometers with similar sensing principles.

## Figures and Tables

**Table 1:** The parameters of the accelerometers.

Accelerometers	Width of Drive Electrodes ( $w_{\mathrm{elec}}$ )	Distance between Pickup and Drive Electrodes ( $d_{\mathrm{elec}}$ )	Position Offset of the Pickup Electrodes ( e−f )	Resonance Frequency
#1	14	6.5	0.9	1216
#2	14	6.0	0.7	1163
#3	14	5.5	1.8	982
Unit	μ m	μ m	μ m	Hz

**Table 2:** Set of frequencies and acceleration amplitudes.

Frequency (Hz)	Amplitude (g)	Frequency Response	Displacement Amplitude of Proof Mass ( μ m)
80	10	1	1.85
160	9.93	1.01	
400	8.98	1.11	
⋯	⋯	⋯	
1220 (near resonance)	1.88	5.32	
⋯	⋯	⋯	
1360	1.92	5.21	
1520	4.50	2.23	

**Table 3:** The optimized parameters of the accelerometers.

Accelerometers	Width of Drive Electrodes ( $w_{\mathrm{elec}}$ )	Distance between Pickup and Drive Electrodes ( $d_{\mathrm{elec}}$ )	Position Offset of the Pickup Electrodes ( e−f )	Resonance Frequency
#4-1	42	6	1	1020
#4-3	42	6	1	1060
Unit	μ m	μ m	μ m	Hz

**Table 4:** VRE performance comparison of the optimized accelerometer with other state-of-the-art MEMS accelerometers.

MEMS Sensors	Bosch Research and Technology Center [31]	Physical Logic [16]	This Work
VRE@10 g	2	4.69	0.55
VRE@20 g	8	14.33	7.2
VRE@30 g	15	20.01	12.61
Unit	mg	mg	mg

## Data Availability

The data that support the findings of this study are available from the corresponding author upon reasonable request. The data are not publicly available due to [institutional policies].
